# Perceived generative AI teaching intelligence and pre-service teachers' professional identity: double-edged pathways through professional agency and AI replacement threat

**DOI:** 10.3389/fpsyg.2026.1953782

**Published:** 2026-09-28

**Authors:** Haoyu Zhao

**Affiliations:** Hubei Communications Research Institute, Hubei Communications Technical College, Wuhan, China

**Keywords:** AI replacement threat, generative artificial intelligence, perceived intelligence, pre-service teachers, teacher education, teacher professional agency, teacher professional identity, teacher–AI complementarity beliefs

## Abstract

Generative artificial intelligence (GenAI) is increasingly used in teacher education for lesson planning, feedback, and assessment support. However, how perceived GenAI teaching intelligence relates to pre-service teachers' professional identity remains unclear. This study examined a double-edged model linking perceived intelligence to professional identity through professional agency and AI replacement threat, with teacher–AI complementarity beliefs moderating these pathways. In a task-based between-subjects experiment, 598 pre-service teachers were randomly assigned to high- or low-intelligence GenAI teaching-support conditions. Complementarity beliefs and baseline professional identity were measured before exposure; perceived intelligence, professional agency, replacement threat, and post-task identity were assessed afterward. Confirmatory factor analysis and conditional process analyses with 5,000 bootstrap resamples tested the model. Participants in the high-intelligence condition reported greater agency and replacement threat, but no statistically significant between-condition difference in post-task identity was detected. Perceived intelligence was positively associated with both agency and replacement threat, which were, respectively, positively and negatively associated with professional identity. Stronger complementarity beliefs were associated with a stronger agency pathway and a weaker replacement-threat pathway. These findings are consistent with two opposing identity-relevant processes following exposure to capable GenAI. Teacher education should support professional judgment and clarify human–AI role boundaries when integrating GenAI into teaching practice.

## 1 Introduction

Generative artificial intelligence (GenAI) is entering the core of teachers' work. Systems based on large language models can draft lesson plans, generate explanations and classroom activities, support learner diagnosis, construct assessment items, and provide feedback (Kasneci et al., 2023; Feuerriegel et al., 2024; Lee and Zhai, 2024). These capabilities have implications beyond instructional efficiency. Because lesson design, diagnosis, and feedback are activities through which teaching expertise is recognized, GenAI may change how future teachers evaluate the value and boundaries of the profession. These questions are particularly consequential for pre-service teachers, whose professional identity develops through coursework, practicum, interactions with teacher educators, and anticipated entry into teaching (Akkerman and Meijer, 2011; Izadinia, 2013). Teacher preparation encompasses subject knowledge and digital competence alongside pedagogical reasoning, ethical responsibility, relational care, and commitment to students' development. Encounters with capable GenAI therefore raise questions about what future teachers can contribute, which responsibilities must remain human, and what makes teaching a distinctive and worthwhile profession.

One interpretation emphasizes augmentation. When GenAI is perceived as understanding instructional needs and providing contextually relevant support, it may expand the options available for planning, reflection, and adaptation. If pre-service teachers retain authority to evaluate, revise, reject, and take responsibility for AI-generated suggestions, this support may strengthen their sense of being able to act as teachers. This reasoning accords with ecological accounts of professional agency and evidence linking agency, AI integration, and language-teacher identity among in-service teachers (Eteläpelto et al., 2013; Priestley et al., 2015; Zou et al., 2026). A second interpretation emphasizes substitution. By performing tasks associated with teaching expertise, the same system may make AI–teacher overlap more salient and provoke doubts about the continuing necessity, recognition, or value of human teaching. These concerns involve occupational substitution and professional devaluation rather than general technology anxiety. Replacement pressure has been documented among pre-service teachers, while research in other professional settings suggests that trainees can experience identity threat when AI appears to encroach on a profession's expertise (Jussupow et al., 2022; Duc et al., 2025).

Recent research supports an ambivalent account of GenAI's professional implications. Double-edged models identify parallel pathways involving efficacy and conflict, self-efficacy and weakened school membership, challenge and threat appraisals, or psychological availability and work alienation (Luo et al., 2025; Ma et al., 2026; Kong et al., 2026; Sun et al., 2026). Teacher-identity studies likewise describe opportunities for creativity, confidence, and pedagogical mediation alongside concerns about authenticity, role uncertainty, and diminished authority (Lan, 2024; Satvati et al., 2025; Haghi et al., 2026; Li and Zhang, 2026; Tümen Akyildiz, 2026). Qualitative evidence from pre-service teachers also documents perceived affordances alongside concerns about dependence, bias, accountability, and AI's place in future practice (Bae et al., 2024; Guan et al., 2025). Evidence from other educational systems places these tensions within professional practice. A UK university study examined teacher agency in relation to student agency and uncertainty surrounding language-model use (Kahn et al., 2025). An Australian formative intervention documented student teachers' agency while evaluating GenAI outputs and negotiating pedagogical decisions (Yang and Markauskaite, 2025). However, three gaps remain. Zou et al. (2026) identify a positive agency mechanism but do not examine a simultaneous identity-threatening pathway. Existing double-edged models primarily address creativity, school adjustment, innovative teaching, or work engagement, whereas teacher-identity studies provide limited quantitative evidence on the coexistence of opposing pathways. It also remains unclear which pre-existing role beliefs shape whether displayed AI capability is interpreted as complementary or substitutive.

To address these gaps, this study develops a model of two opposing pathways from a shared capability appraisal. Perceived intelligence of GenAI in teaching (PIGT) is conceptualized as an identity-relevant appraisal of displayed capability, distinct from acceptance or perceived usefulness. An augmentation interpretation suggests that PIGT may support professional identity through stronger teacher professional agency; a substitution interpretation suggests a negative pathway through AI replacement threat. Teacher–AI complementarity beliefs are proposed to shape these interpretations by specifying whether teachers and AI make differentiated but mutually supportive contributions to education. Stronger complementarity beliefs should help pre-service teachers incorporate AI support into pedagogical action while retaining responsibility for educational decisions, strengthening the agency pathway and attenuating the replacement-threat pathway.

The study makes four contributions. First, it extends research on GenAI in teacher education beyond adoption and task performance to professional self-evaluation. Second, it integrates professional agency and occupational threat within one model to examine their opposing associations with the emerging teacher identity. Third, it introduces teacher–AI complementarity beliefs as a boundary condition grounded in role allocation, distinct from general trust, literacy, or favorable attitudes. Fourth, it combines randomized exposure to standardized teaching support with pre-task measurement of complementarity beliefs and baseline identity, post-task measurement of the focal constructs, condition-level comparisons, and bootstrapped sensitivity analyses. This design distinguishes responses to randomized exposure from associations among measured appraisals and captures immediate identity-relevant responses rather than durable identity change.

## 2 Theoretical framework and hypothesis development

### 2.1 Theoretical framework

The automation–augmentation perspective explains how intelligent technologies can both perform professionally meaningful tasks and expand human capabilities through collaboration (Raisch and Krakowski, 2021). Their professional implications depend partly on how people interpret changes in expertise, responsibility, and role boundaries (Jarrahi, 2018; Faraj et al., 2018; Dellermann et al., 2019). This perspective is relevant to teacher education because professional identity develops through interpretations of competence, responsibility, recognition, and occupational meaning (Beijaard et al., 2004; Akkerman and Meijer, 2011; Izadinia, 2013).

To explain how these interpretations relate to professional identity, the present model specifies professional agency and AI replacement threat as parallel pathways. Professional agency concerns the capacity to shape practice through judgment, decision-making, and purposeful action within contextual conditions (Biesta and Tedder, 2007; Eteläpelto et al., 2013; Priestley et al., 2015). AI replacement threat concerns the perceived vulnerability of valued professional tasks and expertise to technological substitution (Frey and Osborne, 2017; Brougham and Haar, 2018; Jussupow et al., 2022). These pathways extend the broader double-edged logic examined across educational and work contexts to professional identity in teacher preparation (Luo et al., 2025; Ma et al., 2026; Kong et al., 2026; Sun et al., 2026).

Human–AI complementarity perspectives further suggest that these interpretations depend on how responsibilities are allocated between people and intelligent systems (Jarrahi, 2018; Dellermann et al., 2019; Raisch and Krakowski, 2021). In education, teacher–AI complementarity beliefs concern whether AI assistance can extend teaching capacity while preserving teachers' distinctive responsibilities (Holstein et al., 2019; Holstein and Aleven, 2022). They therefore provide a theoretical basis for expecting variation in the agency and replacement-threat pathways at a given level of PIGT.

### 2.2 Hypothesis development

#### 2.2.1 Perceived intelligence of GenAI in teaching, teacher professional agency, and teacher professional identity

PIGT reflects pre-service teachers' perception that GenAI understands instructional contexts, offers relevant suggestions, and supports pedagogical tasks. Teacher professional agency concerns intentional choices, professional judgment, and the capacity to shape educational practice (Eteläpelto et al., 2013; Priestley et al., 2015). From an ecological perspective, access to technological resources alone does not establish agency; teachers must be able to incorporate those resources into their own practice (Biesta and Tedder, 2007; Eteläpelto et al., 2013). Higher PIGT may therefore support agency when AI-generated suggestions become usable resources for decisions that remain under teachers' control.

Two related processes explain this proposed association. First, perceiving GenAI as a source of contextually appropriate support may broaden the options considered for lesson design, learner analysis, and instructional adjustment, consistent with accounts of its educational affordances (Kasneci et al., 2023; Lee and Zhai, 2024). These options should support agency when teachers retain authority to evaluate, modify, and use them (Jarrahi, 2018; Raisch and Krakowski, 2021). Second, retaining control over these choices may strengthen professional authorship: teachers remain the actors who define problems, make decisions, and assume responsibility for outcomes (Eteläpelto et al., 2013; Priestley et al., 2015). Such experiences may reinforce a sense of competence and responsibility central to professional identity (Beijaard et al., 2004; Akkerman and Meijer, 2011; Izadinia, 2013). Evidence linking professional agency, AI integration, and teacher identity is consistent with this proposed positive pathway (Zou et al., 2026).

Accordingly, the following hypothesis is proposed:

**H1**. Perceived intelligence of GenAI in teaching has a positive indirect association with pre-service teachers' professional identity through teacher professional agency.

#### 2.2.2 Perceived intelligence of GenAI in teaching, AI replacement threat, and teacher professional identity

PIGT may also raise concerns about whether human teaching expertise remains distinctive and valuable. AI replacement threat refers to concerns that technological advances may substitute for valued professional tasks, diminish recognition of human expertise, or reduce an occupation's future value (Brougham and Haar, 2018; Frey and Osborne, 2017). It concerns occupational substitution and professional devaluation, distinguishing it from general AI anxiety or resistance to technology. Higher PIGT may therefore be associated with greater replacement threat when GenAI is perceived as capable of performing tasks central to teaching expertise.

This pathway connects perceived task overlap with the value of the future professional self. Lesson planning, explanation, learner diagnosis, assessment, and feedback are visible expressions of teachers' knowledge and judgment. When GenAI appears capable of these activities, pre-service teachers may use its performance as a reference point for evaluating the distinctiveness and necessity of the expertise they are developing (Jussupow et al., 2022; Duc et al., 2025). Research on AI-related professional change similarly identifies opportunities for improvement alongside role uncertainty and replacement pressure (Kong et al., 2026). Such concerns may weaken professional identity because identification with an occupation depends partly on perceiving it as meaningful, socially recognized, and worthy of long-term commitment (Beijaard et al., 2004; Izadinia, 2013). For pre-service teachers, uncertainty about the future value of teaching expertise may therefore undermine anticipated belonging and commitment to the profession (Lan, 2024; Haghi et al., 2026; Tümen Akyildiz, 2026).

Accordingly, the following hypothesis is proposed:

**H2**. Perceived intelligence of GenAI in teaching has a negative indirect association with pre-service teachers' professional identity through AI replacement threat.

#### 2.2.3 Moderating role of teacher–AI complementarity beliefs in the agency pathway

The proposed agency pathway depends on how pre-service teachers understand the relationship between AI assistance and teacher responsibility. Teacher–AI complementarity beliefs concern the differentiated but mutually supportive capabilities of AI and human teachers (Jarrahi, 2018; Dellermann et al., 2019; Raisch and Krakowski, 2021). Unlike general trust or favorable attitudes toward technology, these beliefs emphasize the allocation of roles, authority, and responsibility (Seeber et al., 2020; Glikson and Woolley, 2020). They may therefore shape whether perceived AI capability becomes a resource for professional action.

When complementarity beliefs are strong, pre-service teachers may interpret intelligent GenAI as supporting information processing, content generation, and instructional exploration while retaining responsibility for educational goals, output evaluation, and final pedagogical decisions (Jarrahi, 2018; Holstein et al., 2019; Holstein and Aleven, 2022). This division of responsibilities should make AI-generated options easier to incorporate into professional practice while preserving authorship and control. Because agency entails both access to resources and the capacity to act on them through one's own judgment, higher PIGT should then be more strongly associated with agency (Eteläpelto et al., 2013; Priestley et al., 2015). When complementarity beliefs are weak, capable GenAI may instead be perceived as occupying the same functional space as teachers. Pre-service teachers may consequently find it harder to attribute successful teaching decisions to their own contribution, weakening the proposed PIGT–agency association.

Accordingly, the following hypothesis is proposed:

**H3**. Teacher–AI complementarity beliefs positively moderate the relationship between perceived intelligence of GenAI in teaching and teacher professional agency, such that the positive association is stronger when teacher–AI complementarity beliefs are higher.

#### 2.2.4 Moderating role of teacher–AI complementarity beliefs in the replacement-threat pathway

Complementarity beliefs may also limit the extent to which PIGT is associated with replacement threat. They provide a framework for distinguishing task performance from responsibility for the broader professional role (Jarrahi, 2018; Dellermann et al., 2019; Raisch and Krakowski, 2021). Pre-service teachers who view human and AI contributions as complementary may be less likely to equate the performance of particular instructional tasks with the replacement of teachers (Holstein et al., 2019; Holstein and Aleven, 2022).

Strong complementarity beliefs place AI capabilities within teaching processes that also require contextual judgment, ethical responsibility, relationships with learners, and accountability for educational consequences (Holstein and Aleven, 2022; Oh and Ahn, 2024; Bae et al., 2024). From this perspective, higher PIGT may signal more capable assistance without implying that human expertise is unnecessary. This distinction matters because replacement threat involves an inference from technological capability to diminished professional distinctiveness and future value (Brougham and Haar, 2018; Frey and Osborne, 2017; Jussupow et al., 2022). Complementarity beliefs may weaken that inference by separating the ability to perform part of the work from the ability to assume the professional role. When such beliefs are weak, perceived task overlap may more readily evoke competition and occupational substitution, strengthening the association between PIGT and replacement threat (Duc et al., 2025; Kong et al., 2026).

Accordingly, the following hypothesis is proposed:

**H4**. Teacher–AI complementarity beliefs negatively moderate the relationship between perceived intelligence of GenAI in teaching and AI replacement threat, such that the positive association is weaker when teacher–AI complementarity beliefs are higher.

The proposed framework thus links PIGT to professional identity through two opposing pathways: professional agency and AI replacement threat. Teacher–AI complementarity beliefs are expected to strengthen the PIGT–agency association and weaken the PIGT–replacement-threat association. Figure 1 presents the proposed moderated parallel-mediation model.

**Figure 1:**
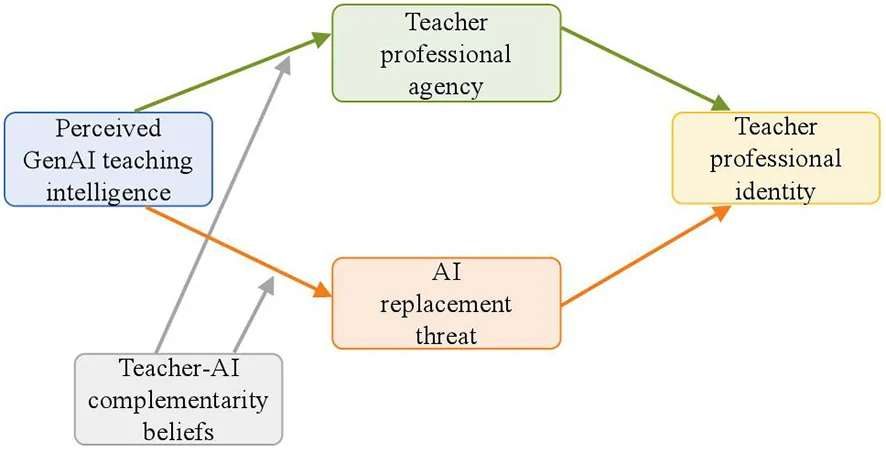
Theoretical model developed in the study.

## 3 Materials and methods

### 3.1 Design and participants

This study combined a task-based between-subjects experiment with pre- and post-task questionnaires. Participants were recruited through teacher-education courses at 41 universities offering recognized teacher-preparation programs in China. Eligible participants were pre-service teachers enrolled in instructional design, curriculum and pedagogy, educational technology, or teaching-practicum preparation courses. The Research Ethics Committee of Hubei Communications Research Institute, China, reviewed the study and granted exemption from full committee review for minimal-risk educational and behavioral research. Participants provided written or electronic informed consent. All participants received the same Grade seven teaching-design brief and the same required deliverables. Random assignment determined which standardized GenAI teaching-support output participants received: the high-intelligence version supplied context-sensitive, specific, and coherently sequenced guidance, whereas the low-intelligence version remained comprehensible but generic and weakly connected. Experimental condition was therefore the randomized exposure; PIGT was the measured post-task appraisal of that capability and served as the focal predictor in the conditional process model. Participants used the assigned output as advisory input while retaining responsibility for their lesson design. Baseline professional identity and teacher–AI complementarity beliefs were measured before exposure; PIGT, professional agency, replacement threat, and post-task professional identity were measured after the task. Figure 2 summarizes the common task, the randomized stimulus contrast, and the measurement sequence. Condition-level comparisons estimated the consequences of randomized exposure, whereas the conditional process analyses estimated theoretically ordered associations among perceived intelligence, agency, replacement threat, and post-task identity.

**Figure 2:**
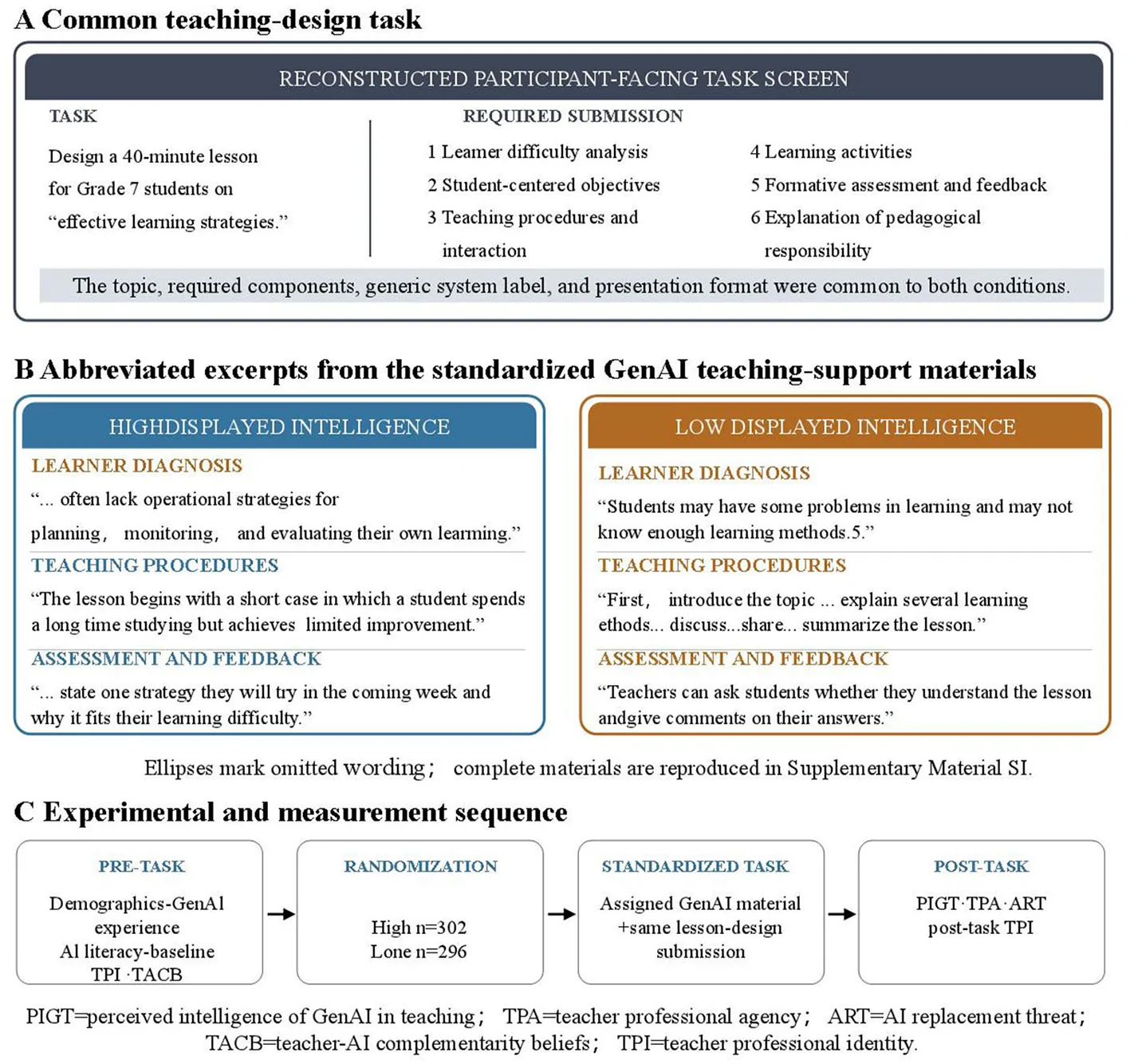
Teaching-design task, experimental manipulation, and measurement sequence.

### 3.2 Procedure and data screening

The formal study was administered online via Wenjuanxing between 2025-06-01 and 2025-07-15. The procedure followed a fixed sequence (Figure 2). Participants first completed demographic questions, prior GenAI experience, perceived AI literacy, baseline professional identity, and teacher–AI complementarity beliefs. They were then randomly assigned to the high- or low-intelligence teaching-support condition. Random assignment was implemented automatically by the survey platform's randomization function. The common task instruction stated that the AI teaching-assistant output could be used as a reference but that the final teaching design should reflect the participant's own professional judgment. Participants reviewed the assigned output and completed the same structured lesson-design submission. Completion of the six-component submission was required before the post-task questionnaire could be accessed, ensuring engagement with the teaching-design task. After the task, they completed the PIGT manipulation-check items and the measures of professional agency, replacement threat, and post-task professional identity. Complementarity beliefs were measured before exposure because they were theorized as a pre-existing interpretive schema. The research team screened for duplicate participant IDs, missing analyzed values, failed attention checks, invariant response patterns, and unusually short completion times. Of the 667 responses received, 69 were excluded by these checks; the remaining 598 complete cases were retained, with 302 participants assigned to the high-intelligence condition and 296 to the low-intelligence condition.

### 3.3 Teaching-design task and experimental manipulation

The participant-facing task asked each pre-service teacher to design a 40-min lesson for Grade seven students on “effective learning strategies.” This cross-disciplinary topic was selected to reduce differences in subject knowledge while preserving core teaching practices, including diagnosing learner needs, setting objectives, designing activities, and planning feedback. Participants completed a structured template covering six components: learner difficulty analysis, student-centered objectives, teaching procedures and interaction, learning activities, formative assessment and feedback, and teachers' pedagogical responsibility. The common instruction permitted reference to the AI materials while assigning participants responsibility for the final pedagogical decisions. Figure 2A presents the task interface.

Two AI teaching-support materials were developed from the same prompt and presented using the same topic, duration, structure, label, and visual format. The manipulation focused on the level of displayed teaching intelligence, reflected in contextual understanding, pedagogical specificity, instructional coherence, and feedback relevance. Thus, the materials represented a bundled contrast in displayed teaching intelligence rather than the effect of any single textual feature. The high-intelligence material presented more specific pedagogical guidance by identifying specific learner difficulties, organizing learning strategies into planning, active processing, and reflection, and linking classroom activities with formative assessment. In contrast, the low-intelligence material provided topic-relevant but general suggestions, with limited diagnosis, instructional specificity, and feedback guidance. Figure 2B provides representative excerpts from both materials.

Three experts in teacher education and educational technology reviewed the materials for the intended intelligence contrast and comparability in topic, structure, readability, and format. A pilot study with 54 pre-service teachers assessed the clarity of the materials and the perceived-intelligence contrast and led to minor revisions. After completing the task, participants rated whether the GenAI teaching assistant understood instructional needs, recognized teaching contexts, provided pedagogically relevant information, and understood teachers' intentions. An independent-samples *t test* compared PIGT between the randomized conditions. These measured appraisals served as the focal predictor in the conditional process model.

### 3.4 Measures and scale adaptation

All focal items used five-point response scales ranging from 1 (strongly disagree) to 5 (strongly agree). English-language items were translated into Chinese by two bilingual researchers and independently back-translated by a third researcher. Discrepancies were reconciled by the research team. Three experts in teacher education and educational technology then reviewed conceptual coverage, contextual appropriateness, and wording, and the pilot test assessed item clarity and response variability.

PIGT was assessed with four items adapted from perceived-intelligence measures in human–AI interaction and GenAI research (Bartneck et al., 2009; Luo et al., 2025). The items assessed whether the GenAI teaching assistant understood instructional needs, recognized the teaching context, provided pedagogically relevant information, and appeared to understand teachers' instructional intentions.

Teacher professional agency (TPA) was assessed with five context-adapted items informed by professional-agency theory (Eteläpelto et al., 2013; Priestley et al., 2015) and the teacher-agency domains identified by Leijen et al. (2024). The items captured professional judgment, initiative, adaptation of AI-generated suggestions to learner needs, and retention of human control and responsibility in AI-supported teaching.

AI replacement threat (ART) was assessed with four items adapted from research on occupational threat associated with smart technology, artificial intelligence, robotics, and algorithms and recent work with pre-service teachers (Brougham and Haar, 2018; Duc et al., 2025). The items addressed concern that GenAI could replace important teaching tasks, reduce the need for human teachers, weaken the future value of teaching, or take over teachers' work.

Teacher–AI complementarity beliefs (TACB) were measured before exposure with five items developed for this study from research on human–AI complementarity and AI-augmented teaching (Jarrahi, 2018; Holstein et al., 2019; Holstein and Aleven, 2022; Raisch and Krakowski, 2021). The items assessed whether AI and teachers were viewed as making mutually supportive rather than substitutive contributions. This study-specific scale underwent preliminary content and structural validation.

The five complementarity items were reviewed by the three experts for relevance, conceptual coverage, and clarity. In the pilot sample, all items loaded on one factor, with loadings from 0.72 to 0.86, and the factor explained 62.4% of the variance. Reliability, convergent validity, and discriminant validity were then evaluated in the formal sample. The scale should nevertheless be independently validated in additional samples.

Teacher professional identity (TPI) was assessed with five items adapted from pre-service teacher identity research, particularly role recognition, professional value, affective attachment, and career commitment (Beijaard et al., 2004; Hong, 2010; Hanna et al., 2020). The same items were administered before and after the task. Baseline identity was the mean of the five pre-task items (*M* = 3.799, *SD* = 0.646, Cronbach's α = 0.889); post-task identity was the mean of the five post-task items and served as the focal outcome (*M* = 3.833, *SD* = 0.625, Cronbach's α = 0.885).

Gender, grade, prior GenAI experience, perceived AI literacy, and baseline professional identity were examined in sensitivity analyses. Condition was coded 0 = low intelligence and 1 = high intelligence; gender was coded 0 = man and 1 = woman; and grade was represented by three dummy variables, with first-year students as the reference group. Prior GenAI experience was measured with one five-point frequency item. Perceived AI literacy was the mean of three items assessing the ability to understand, evaluate, and use AI tools (*M* = 3.466, *SD* = 0.749, Cronbach's α = 0.826). AI literacy and baseline identity entered the analyses as observed mean scores. Major was recorded descriptively but was not included as a covariate because the programs represented heterogeneous nominal specializations. Table 1 reports the focal items.

**Table 1:** Construct measures used in the study.

Construct	Code	Item
Perceived intelligence of GenAI in teaching	PIGT1	The GenAI teaching assistant understands instructional needs well.
	PIGT2	The GenAI teaching assistant recognizes the context of teaching tasks.
	PIGT3	The GenAI teaching assistant provides pedagogically relevant information needed for teaching.
	PIGT4	The GenAI teaching assistant appears to understand teachers' instructional intentions.
Teacher professional agency	TPA1	I can make my own professional judgments when using AI-supported teaching resources.
	TPA2	I can adapt AI-generated teaching suggestions according to students' learning needs.
	TPA3	I can take initiative in designing teaching activities in an AI-supported classroom.
	TPA4	I can use AI-generated information while still maintaining my professional role as a future teacher.
	TPA5	I can decide when and how AI should be used in teaching practice.
AI replacement threat	ART1	GenAI makes me worry that some important teaching tasks may be replaced by AI.
	ART2	The development of GenAI makes me uncertain about the future value of human teachers.
	ART3	GenAI may reduce the need for teachers in some teaching-related tasks.
	ART4	I feel pressure when thinking about the possibility that AI may take over teachers' work.
Teacher–AI complementarity beliefs	TACB1	AI can assist teachers, but it cannot replace teachers' educational responsibility.
	TACB2	AI can improve teaching efficiency, but teachers remain essential for guiding students' growth.
	TACB3	The development of AI makes teachers' professional judgment more important, not less important.
	TACB4	Future teachers should learn to work with AI rather than compete with AI.
	TACB5	AI is better understood as a teaching partner than as a substitute for teachers.
Teacher professional identity	TPI1	I identify with the role of being a teacher.
	TPI2	I believe teaching is a meaningful profession.
	TPI3	I feel proud of being trained as a future teacher.
	TPI4	I am willing to become a teacher in the future.
	TPI5	Becoming a teacher is an important part of my future career plan.

PIGT, perceived intelligence of GenAI in teaching; TPA, teacher professional agency; ART, AI replacement threat; TACB, teacher–AI complementarity beliefs; TPI, teacher professional identity. All items were measured on a five-point Likert scale ranging from 1 = strongly disagree to 5 = strongly agree.

### 3.5 Data analysis

Analyses were conducted in IBM SPSS Statistics 27, IBM SPSS Amos 27, and Python 3.13.5. Preliminary analyses included data-quality screening, descriptive statistics, zero-order correlations, randomization checks, and manipulation checks. An analysis of covariance (ANCOVA) was conducted separately to examine post-task professional identity while controlling for baseline identity. All inferential tests were two-tailed. A sensitivity power analysis (G^*^Power 3.1; two-tailed α = 0.05) indicated that the sample of 598 participants provided 80% power, under individual-level regression assumptions, to detect incremental effects as small as f^2^ = 0.013 in the focal regression equations, below the smallest significant interaction effect observed (f^2^ = 0.014). University defined the institutional nesting of participants. Potential within-university dependence was examined descriptively. The 41 clusters ranged from 5 to 38 participants (median = 14). Unconditional random-intercept models yielded intraclass correlations of 0.06, 0.08, and 0.07 for agency, threat, and post-task identity, respectively. Anonymity, neutral instructions, and pre-task measurement of complementarity beliefs and baseline identity were used to reduce common-method risk. Given that several constructs were assessed in the same post-task session, supplementary diagnostics including Harman's single-factor test, full-collinearity variance inflation factors (VIFs), and an Amos common latent factor comparison were conducted (Podsakoff et al., 2003; Kock, 2017). The five-factor measurement model was estimated by maximum likelihood confirmatory factor analysis. Model fit was evaluated using χ^2^/df, the comparative fit index (CFI), the Tucker–Lewis index (TLI), the root mean square error of approximation (RMSEA) and its 90% confidence interval (CI), and the root mean square residual (RMR) (Hu and Bentler, 1999). Standardized loadings, Cronbach's alpha, composite reliability, and average variance extracted were examined to assess internal consistency and convergent validity (Fornell and Larcker, 1981). Heterotrait-monotrait (HTMT) ratios below 0.85 were used as evidence of discriminant validity (Henseler et al., 2015).

After acceptable measurement quality was established, conditional process models were estimated by ordinary least squares (OLS) using standardized scale scores. Professional agency and replacement threat were regressed on PIGT, teacher–AI complementarity beliefs, and their product term; post-task professional identity was regressed on PIGT, agency, and replacement threat. Predictor VIFs, R^2^, and f^2^ effect sizes were examined. Specific indirect and moderated-indirect associations were estimated with 5,000 nonparametric participant-level bootstrap resamples and percentile 95% confidence intervals. Interactions were probed at one standard deviation below and above the complementarity-belief mean (Hayes, 2022). H1 and H2 were evaluated with the two specific bootstrap indirect associations at mean complementarity beliefs. H3 and H4 were evaluated with the first-stage interaction terms, supplemented by the indices of moderated mediation and conditional indirect associations. The direct PIGT–identity association was estimated without a directional hypothesis. The primary model included only the theoretical variables. Sensitivity specifications added condition to all three endogenous equations, omitted the direct path, added demographic and AI-experience covariates, added baseline identity to the fully adjusted equations, and used baseline-residualized post-task identity as the outcome. No latent-mean or structural-path comparison was conducted between conditions.

## 4 Results

### 4.1 Data screening and preliminary checks

Of the 667 responses received, 69 were excluded during screening for duplicate IDs, missing analyzed values, failed attention checks, invariant response patterns, or unusually short completion times; 598 complete cases were retained: 302 in the high-intelligence condition and 296 in the low-intelligence condition. Women represented 72.7% of the sample (*n* = 435). First-, second-, third-, and fourth-year students represented 20.1%, 29.8%, 32.3%, and 17.9% of the sample, respectively. The conditions did not differ significantly at baseline in professional identity, *t*(596) = 0.773, *p* = 0.440; perceived AI literacy, *t*(596) = −1.136, *p* = 0.256; prior GenAI experience, *t*(596) = 0.971, *p* = 0.332; gender distribution, continuity-corrected χ^2^(1) = 0.161, *p* = 0.689; grade distribution, χ^2^(3) = 5.292, *p* = 0.152; or major distribution, χ^2^(7) = 6.714, *p* = 0.459. No statistically detectable baseline imbalances were observed in these measured characteristics. Beyond gender and grade, the sample covered eight teacher-preparation program categories, including primary education, preschool education, Chinese language education, English education, mathematics education, science education, and educational technology. Participants reported moderate prior experience with GenAI tools for learning or teaching-related tasks (*M* = 3.21, *SD* = 0.96) and moderate-to-high perceived AI literacy (*M* = 3.47, *SD* = 0.75) on the five-point scales.

### 4.2 Manipulation check

In the independent pilot sample (*N* = 54), perceived-intelligence ratings were higher for the high-intelligence material (*M* = 3.84, *SD* = 0.59) than for the low-intelligence material (*M* = 3.08, *SD* = 0.61), *t*(52) = 4.66, *p* < 0.001, Cohen's *d* = 1.27, supporting the intended contrast. The formal manipulation check corroborated this pattern: participants in the high-intelligence condition reported higher PIGT (*M* = 3.88, *SD* = 0.72) than those in the low-intelligence condition (*M* = 2.94, *SD* = 0.70), *t*(596) = 16.27, *p* < 0.001, Cohen's *d* = 1.33. The manipulation therefore produced a large difference in perceived teaching intelligence.

### 4.3 Condition-level outcome checks

Participants in the high-intelligence condition reported higher post-task professional agency (*M* = 3.70, *SD* = 0.73) than those in the low-intelligence condition (*M* = 3.43, *SD* = 0.68), *t*(596) = 4.666, *p* < 0.001, *d* = 0.382. They also reported higher replacement threat (*M* = 3.11, *SD* = 0.82 vs. *M* = 2.88, *SD* = 0.75), *t*(596) = 3.522, *p* < 0.001, *d* = 0.288. The difference in post-task professional identity was not statistically significant (*M* = 3.87, *SD* = 0.63 vs. *M* = 3.80, *SD* = 0.61), *t*(596) = 1.467, *p* = 0.143, *d* = 0.120. ANCOVA adjusting for baseline identity yielded the same pattern, *F*(1, 595) = 1.574, *p* = 0.210, partial η^2^ = 0.003; adjusted means were 3.858 and 3.807, respectively. Thus, exposure to the high-intelligence material elicited higher scores on both proposed mediators, without a statistically detectable difference in post-task identity.

### 4.4 Descriptive statistics and correlations

Table 2 presents descriptive statistics and zero-order correlations. PIGT correlated positively with professional agency (*r* = 0.345, *p* < 0.01) and replacement threat (*r* = 0.217, *p* < 0.01). Professional agency correlated positively with post-task professional identity (*r* = 0.477, *p* < 0.01), whereas replacement threat correlated negatively with it (*r* = −0.313, *p* < 0.01). Complementarity beliefs correlated positively with agency and identity and negatively with replacement threat. This pattern was consistent with the proposed double-edged structure: PIGT was positively related to both proximal responses, which had opposite-signed associations with post-task identity. None of the correlations suggested substantial construct overlap.

**Table 2:** Descriptive statistics and correlations.

Variable	M	SD	1	2	3	4	5	6	7	8
1. GenAI exp.	3.210	0.958	—							
2. AI literacy	3.466	0.749	0.298^**^	—						
3. Baseline TPI	3.799	0.646	0.143^**^	0.188^**^	—					
4. PIGT	3.415	0.855	0.233^**^	0.135^**^	0.131^**^	—				
5. TPA	3.563	0.717	0.245^**^	0.245^**^	0.287^**^	0.345^**^	—			
6. ART	2.999	0.794	−0.077	−0.157^**^	−0.196^**^	0.217^**^	−0.053	—		
7. TACB	3.680	0.766	0.159^**^	0.234^**^	0.265^**^	0.186^**^	0.364^**^	−0.264^**^	—	
8. Post TPI	3.833	0.625	0.186^**^	0.270^**^	0.612^**^	0.114^**^	0.477^**^	−0.313^**^	0.431^**^	—

*N* = 598. PIGT, perceived intelligence of GenAI in teaching; TPA, teacher professional agency; ART, AI replacement threat; TACB, teacher–AI complementarity beliefs; TPI, teacher professional identity. ^*^p < 0.05, ^**^p < 0.01.

### 4.5 Common method bias

The first unrotated factor accounted for 31.05% of the total item variance. Full-collinearity VIFs ranged from 1.238 to 1.547. In the Amos common-latent-factor comparison, absolute changes in the 23 standardized trait loadings averaged 0.004 and did not exceed 0.014. These diagnostics did not indicate a dominant common-method factor, although they cannot rule out covariance attributable to same-session measurement.

### 4.6 Measurement model: reliability and validity

The five-factor confirmatory factor model fit the data well, χ^2^(220) = 233.394, *p* = 0.255, χ^2^/df = 1.061, CFI = 0.998, TLI = 0.998, RMSEA = 0.010, 90% CI [0.000, 0.020], and RMR = 0.016. Standardized loadings ranged from 0.765 to 0.840 (Table 3). Cronbach's alpha and composite reliability ranged from 0.877 to 0.904, and average variance extracted ranged from 0.607 to 0.670, supporting internal consistency and convergent validity across the five focal constructs. All heterotrait-monotrait ratios were below 0.85 (Table 4), supporting discriminant validity. Professional agency, complementarity beliefs, and post-task professional identity were therefore empirically distinguishable despite their theoretical relatedness. All measurement analyses used the pooled sample; no between-condition comparisons of latent means or structural paths were conducted.

**Table 3:** Reliability and convergent validity.

Construct/ item	CFA loading	Cronbach's α	CR	AVE
Perceived intelligence of GenAI in teaching		0.890	0.890	0.670
PIGT1	0.823			
PIGT2	0.792			
PIGT3	0.840			
PIGT4	0.819			
Teacher professional agency		0.903	0.903	0.650
TPA1	0.819			
TPA2	0.803			
TPA3	0.809			
TPA4	0.815			
TPA5	0.785			
AI replacement threat		0.877	0.877	0.640
ART1	0.800			
ART2	0.798			
ART3	0.807			
ART4	0.796			
Teacher–AI complementarity beliefs		0.904	0.904	0.653
TACB1	0.786			
TACB2	0.811			
TACB3	0.827			
TACB4	0.795			
TACB5	0.821			
Post-task teacher professional identity		0.885	0.885	0.607
TPI1	0.765			
TPI2	0.788			
TPI3	0.781			
TPI4	0.791			
TPI5	0.769			

CFA loading, standardized confirmatory-factor loading; α, Cronbach's alpha; CR, composite reliability; AVE, average variance extracted; k, number of items. TPI refers to post-task teacher professional identity.

**Table 4:** Heterotrait-monotrait ratio matrix.

Construct	1	2	3	4	5
1. PIGT	—				
2. TPA	0.385	—			
3. ART	0.246	0.060	—		
4. TACB	0.207	0.402	0.296	—	
5. TPI	0.128	0.533	0.354	0.482	—

PIGT, perceived intelligence of GenAI in teaching; TPA, teacher professional agency; ART, AI replacement threat; TACB, teacher–AI complementarity beliefs; TPI, teacher professional identity.

### 4.7 Focal model and hypothesis tests

Predictor VIFs ranged from 1.016 to 1.212, indicating no problematic multicollinearity. The regression equations accounted for 23.2% of the variance in professional agency, 15.5% in replacement threat, and 31.1% in post-task professional identity. Path-specific *f*^2^ values ranged from 0.001 for the direct PIGT–identity association to 0.258 for the agency–identity association.

Figure 3 and Table 5 report standardized estimates from the individual-level OLS models. PIGT was positively associated with professional agency (β = 0.295, *p* < 0.001, 95% CI [0.223, 0.367]) and replacement threat (β = 0.271, *p* < 0.001, 95% CI [0.195, 0.346]). Professional agency was positively associated with post-task identity (β = 0.454, *p* < 0.001, 95% CI [0.382, 0.526]), whereas replacement threat was negatively associated with it (β = −0.293, *p* < 0.001, 95% CI [−0.362, −0.224]). Complementarity beliefs were positively associated with agency (β = 0.325, *p* < 0.001) and negatively associated with replacement threat (β = −0.327, *p* < 0.001). The direct PIGT–identity association was not statistically significant (β = 0.021, *p* = 0.581, 95% CI [−0.053, 0.094]).

**Figure 3:**
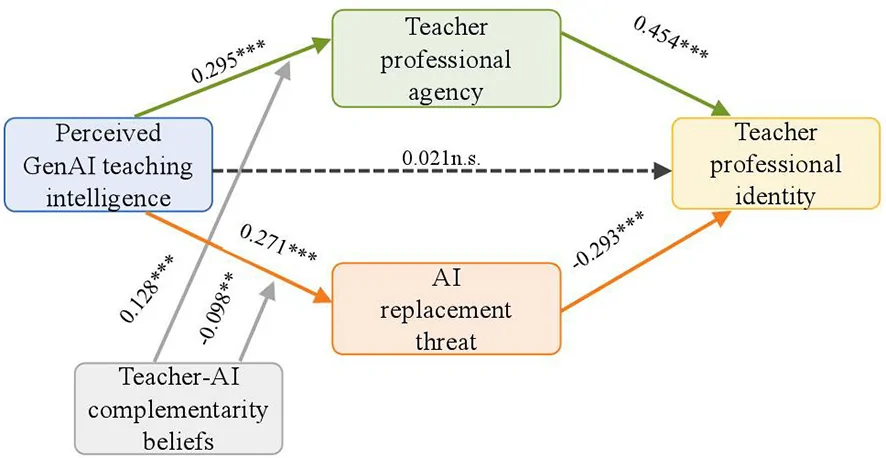
Standardized regression estimates for the moderated parallel-mediation model.

**Table 5:** Standardized regression coefficients for the focal model.

Path	β	SE	*t*	*p*	95% CI
PIGT → TPA	0.295	0.037	8.046	< 0.001	[0.223, 0.367]
PIGT → ART	0.271	0.038	7.038	< 0.001	[0.195, 0.346]
TPA → TPI	0.454	0.037	12.386	< 0.001	[0.382, 0.526]
ART → TPI	−0.293	0.035	−8.318	< 0.001	[−0.362, −0.224]
TACB → TPA	0.325	0.037	8.835	< 0.001	[0.253, 0.397]
TACB → ART	−0.327	0.039	−8.469	< 0.001	[−0.403, −0.251]
PIGT × TACB → TPA	0.128	0.032	3.955	< 0.001	[0.065, 0.192]
PIGT × TACB → ART	−0.098	0.034	−2.871	0.004	[−0.165, −0.031]
PIGT → TPI	0.021	0.038	0.552	0.581	[−0.053, 0.094]

Coefficients are standardized ordinary least squares estimates.

At the mean level of complementarity beliefs, the two specific indirect associations had opposite signs. The indirect association through professional agency was positive and statistically significant (β = 0.134, 95% bootstrap CI [0.100, 0.171]), supporting H1. The indirect association through replacement threat was negative and statistically significant (β = −0.079, 95% bootstrap CI [−0.111, −0.051]), supporting H2. Their algebraic sum yielded a smaller net indirect association (β = 0.055, 95% bootstrap CI [0.007, 0.101]). The conditional total association at mean complementarity beliefs was not statistically significant (β = 0.075, 95% bootstrap CI [−0.005, 0.154]); the double-edged interpretation therefore rests on the oppositely signed specific indirect associations.

The positive indirect point estimate through agency was approximately 1.7 times the magnitude of the negative estimate through replacement threat. Their net estimate remained positive but was smaller than either component in absolute magnitude, consistent with partial cancellation within the fitted model.

The PIGT × complementarity-beliefs interaction was positively associated with professional agency (β = 0.128, *p* < 0.001). The estimated first-stage slope increased from 0.166 at low complementarity beliefs to 0.423 at high complementarity beliefs. The corresponding conditional indirect association through agency increased from 0.076, 95% CI [0.024, 0.129], to 0.192, 95% CI [0.148, 0.241]. The index of moderated mediation was 0.058, 95% CI [0.024, 0.095], supporting H3 in the individual-level model (Table 6).

**Table 6:** Bootstrap decomposition and moderated-mediation estimates.

Association	β/Index	SE	95% CI	Result
PIGT → TPA → TPI	0.134	0.018	[0.100, 0.171]	H1 supported
PIGT → ART → TPI	−0.079	0.016	[−0.111, −0.051]	H2 supported
Net indirect association	0.055	0.024	[0.007, 0.101]	Significant
Direct association: PIGT → TPI	0.021	0.038	[−0.053, 0.094]	Nonsignificant
Conditional total association at mean TACB	0.075	0.039	[−0.005, 0.154]	Nonsignificant; not interpreted
Moderated mediation via TPA	0.058	0.018	[0.024, 0.095]	H3 supported
Low TACB: PIGT → TPA → TPI	0.076	0.027	[0.024, 0.129]	Significant
High TACB: PIGT → TPA → TPI	0.192	0.024	[0.148, 0.241]	Significant
Moderated mediation via ART	0.029	0.012	[0.006, 0.053]	H4 supported (small effect)
Low TACB: PIGT → ART → TPI	−0.108	0.022	[−0.154, −0.069]	Significant
High TACB: PIGT → ART → TPI	−0.051	0.018	[−0.089, −0.017]	Significant

Low and high TACB refer to one standard deviation below and above the mean, respectively. All confidence intervals are percentile 95% intervals based on 5,000 bootstrap resamples.

The PIGT × complementarity-beliefs interaction was negatively associated with replacement threat (β = −0.098, *p* = 0.004). The first-stage slope decreased from 0.368 at low complementarity beliefs to 0.173 at high complementarity beliefs, indicating attenuation without a reversal in sign. The negative conditional indirect association through replacement threat decreased in magnitude from −0.108, 95% CI [−0.154, −0.069], to −0.051, 95% CI [−0.089, −0.017]. The index of moderated mediation was positive (0.029, 95% CI [0.006, 0.053]) because the interaction coefficient and the threat–identity coefficient were both negative. Thus, stronger complementarity beliefs were associated with a less negative indirect association. H4 was supported in the individual-level analysis, although the interaction was small.

Across low and high complementarity beliefs, the estimated first-stage agency slope increased approximately 2.5-fold (from 0.166 to 0.423), while the estimated threat slope decreased by roughly one half (from 0.368 to 0.173). These patterns were directionally complementary: stronger beliefs accompanied a stronger agency-related association and a weaker threat-related association.

Together, these estimates support the proposed pattern of oppositely signed indirect associations between PIGT and post-task professional identity. The nonsignificant direct path does not establish complete mediation. Because identity was assessed immediately after a single teaching-design task, the estimates concern task-specific professional self-appraisals.

### 4.8 Robustness analyses

Table 7 summarizes the sensitivity analyses based on 5,000 participant-level bootstrap resamples. Including randomized condition (0 = low, 1 = high) as a predictor in the equations for professional agency, replacement threat, and post-task identity left both specific indirect associations and both indices of moderated mediation statistically significant. Omitting the nonsignificant direct path also left these statistical conclusions unchanged.

**Table 7:** Sensitivity analyses of indirect and moderated-indirect associations.

Model specification	Indirect via TPA	Indirect via ART	Index via TPA	Index via ART
Primary model	0.134 [0.100, 0.171]	−0.079 [−0.111, −0.051]	0.058 [0.024, 0.095]	0.029 [0.006, 0.053]
Condition added to TPA, ART, and post-task TPI	0.130 [0.090, 0.172]	−0.078 [−0.113, −0.047]	0.058 [0.023, 0.094]	0.029 [0.006, 0.053]
Direct path excluded	0.136 [0.102, 0.173]	−0.078 [−0.108, −0.051]	0.059 [0.024, 0.096]	0.028 [0.005, 0.053]
Demographic, experience, and AI-literacy controls in all equations	0.111 [0.078, 0.147]	−0.077 [−0.108, −0.049]	0.051 [0.020, 0.085]	0.025 [0.004, 0.047]
Fully adjusted, including baseline TPI in all equations	0.081 [0.056, 0.109]	−0.054 [−0.080, −0.032]	0.036 [0.012, 0.062]	0.015 [0.001, 0.032]
Baseline-residualized post-task TPI as outcome	0.113 [0.083, 0.148]	−0.058 [−0.087, −0.033]	0.049 [0.020, 0.080]	0.021 [0.004, 0.042]

Entries are standardized estimates with percentile 95% bootstrap confidence intervals based on 5,000 resamples. All listed confidence intervals exclude zero.

Adding gender, grade, prior GenAI experience, and perceived AI literacy to all three endogenous equations reduced the positive indirect estimate but preserved support for the four focal hypotheses. A fully adjusted specification that also included baseline identity yielded an agency indirect association of 0.081, 95% CI [0.056, 0.109], a replacement-threat indirect association of −0.054, 95% CI [−0.080, −0.032], and statistically significant indices of moderated mediation through agency (0.036, 95% CI [0.012, 0.062]) and replacement threat (0.015, 95% CI [0.001, 0.032]).

A baseline-adjusted sensitivity analysis residualized post-task identity on baseline identity before estimating the theoretical model. The indirect associations through agency (β = 0.113, 95% CI [0.083, 0.148]) and replacement threat (β = −0.058, 95% CI [−0.087, −0.033]) remained statistically significant, as did the indices of moderated mediation through agency (0.049, 95% CI [0.020, 0.080]) and replacement threat (0.021, 95% CI [0.004, 0.042]).

These specifications assess sensitivity to model specification and covariate adjustment; they do not establish causal ordering. The proposed sequence was specified on theoretical grounds, with agency and replacement threat conceptualized as proximal interpretations of perceived GenAI capability. Although exposure was randomized, PIGT, the mediators, and post-task identity were measured in the same session, precluding a firm determination of their temporal order.

Across the individual-level specifications in Table 7, the signs and statistical conclusions for the two specific indirect associations and the two indices of moderated mediation were unchanged. Figure 4 displays the first-stage simple slopes, and Table 6 reports the conditional indirect associations.

**Figure 4:**
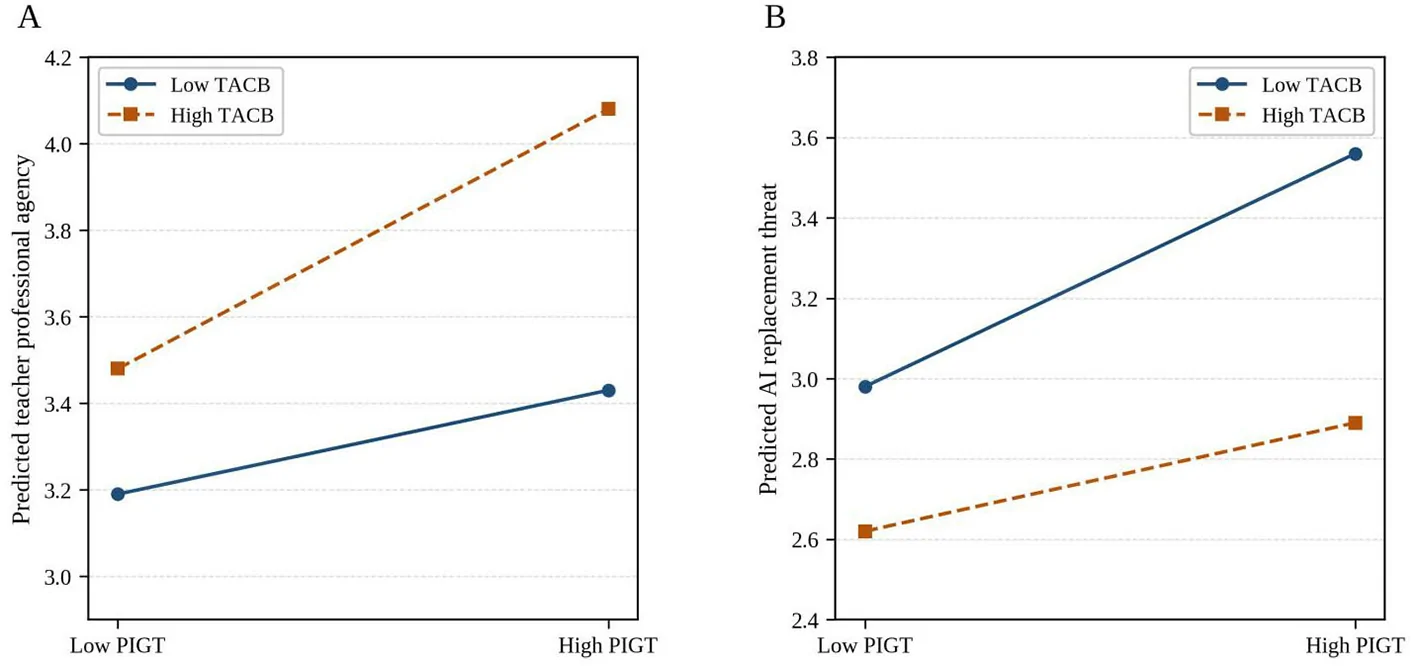
Simple slopes for the moderating effects of teacher–AI complementarity beliefs on **(A)** teacher professional agency and **(B)** AI replacement threat. PIGT = perceived GenAI teaching intelligence; TACB = teacher–AI complementarity beliefs. Low and high values represent one standard deviation below and above the mean. Predicted values are shown on the original five-point scale.

## 5 Discussion

### 5.1 Interpretation of the findings

The findings identify distinct professional responses to GenAI teaching support. Randomized exposure to the high-intelligence material increased reported professional agency and replacement threat, while the condition-level difference in post-task identity was not statistically significant. At the appraisal level, PIGT was linked to identity through two statistically significant indirect associations with opposite signs, and their strength varied with complementarity beliefs. This pattern is consistent with a process account in which the professional meaning of perceived AI capability varies with future teachers' appraisals.

Participants who perceived greater understanding of instructional needs also reported greater capacity to adapt suggestions, initiate action, and decide how AI should be used. This agency-related association suggests that perceived capability can be compatible with an active professional role. The positive association between PIGT and replacement threat is consistent with concern about overlap between AI capabilities and recognizable teacher work; greater replacement threat, in turn, was associated with weaker post-task identity. This pattern aligns with qualitative accounts of authenticity crises, diminished recognition, role uncertainty, and authority renegotiation in AI-mediated teaching (Almashour et al., 2025; Haghi et al., 2026; Tümen Akyildiz, 2026). It also accords with evidence of AI-related threat appraisals among practicing teachers and replacement pressure among pre-service teachers (Duc et al., 2025; Kong et al., 2026). Experimental workplace research also found that AI users anticipated and received less favorable evaluations of competence and motivation (Reif et al., 2025). Although these judgments differ from teachers' own replacement concerns, they suggest that access to technical assistance need not translate into greater professional recognition.

Complementarity beliefs were associated with differences in both proposed pathways: the agency-related indirect association was stronger, and the threat-related indirect association was less negative, at higher belief levels. This dual pattern is theoretically informative because recent studies have examined broader resources such as team trust, AI literacy, and digital competency (Luo et al., 2025; Ma et al., 2026; Sun et al., 2026). Teacher–AI complementarity beliefs address the role relationship more directly by distinguishing coordinated specialization from perceived redundancy. The negative indirect association through threat remained detectable at high complementarity beliefs, suggesting that perceived complementarity and replacement concerns can coexist in the same task.

### 5.2 Theoretical implications

First, the study extends GenAI research in teacher education from adoption and performance to professional self-evaluation. For future teachers, perceived AI capability bears on three identity questions: what can I do as a teacher, what remains distinctively human in teaching, and is the profession still worth entering? Examining immediate professional self-appraisal connects human–AI interaction with the dialogical and developmental psychology of teacher preparation (Akkerman and Meijer, 2011).

Second, the study specifies how a common capability cue can be associated with opposing professional appraisals. Perceived teaching intelligence was related to identity through a positive agency-related indirect association and a negative threat-related indirect association. This account extends double-edged GenAI research beyond team creativity and school adjustment (Luo et al., 2025; Ma et al., 2026; Kong et al., 2026; Sun et al., 2026) by identifying associations relevant to professional value and identity in an occupation defined by social and ethical responsibilities.

Third, the study integrates perspectives on professional agency and identity threat. Agency encompasses evaluating AI-generated suggestions, adapting them to learners, deciding when AI should be used, and retaining responsibility for educational consequences (Lan, 2024; Satvati et al., 2025; Haghi et al., 2026; Zou et al., 2026). It therefore extends beyond proficiency in tool use. Replacement threat captures concern that task overlap may diminish professional recognition and anticipated occupational value. Considering these appraisals together helps explain how capable AI can be experienced as both a professional resource and a challenge to the future teacher's role.

Fourth, teacher–AI complementarity beliefs are conceptualized as a schema for interpreting professional role boundaries. They concern how human and artificial capacities should be distributed within teaching, which distinguishes them conceptually from AI literacy, self-efficacy, trust, and general attitudes. The observed moderation pattern is consistent with the proposition that the professional meaning of perceived AI capability varies with beliefs about how human and artificial agency should coexist. This construct thus connects human–AI role design with professional identity at the level of individual appraisal.

### 5.3 Practical implications

Teacher-education programs should situate GenAI competence within pedagogical professionalism. Research with Finnish pre-service teachers links AI-specific pedagogical knowledge and advanced prompting strategies with more adaptive lesson plans, reinforcing the importance of pedagogical reasoning in AI preparation (Celik et al., 2026). Students need opportunities to evaluate factual accuracy, pedagogical fit, bias, privacy, ethical acceptability, relational consequences, and responsibility for subsequent decisions. The findings motivate testing training approaches that combine AI literacy with explicit reflection on professional judgment and role boundaries.

AI-supported assignments can make professional judgment visible at key stages. Students can diagnose learner needs before prompting, compare alternative outputs, identify unsupported assumptions, revise suggestions for a defined classroom context, document what they accept or reject, and justify the final pedagogical decision. Assessment rubrics should recognize diagnosis, justification, adaptation, and accountability alongside the quality of the completed materials.

Replacement concerns warrant explicit discussion in teacher education. Role-boundary mapping can distinguish tasks suitable for automation, tasks requiring human–AI collaboration, and responsibilities that remain primarily human because they involve care, value judgment, inclusion, contextual interpretation, or accountability. Practicum reflection and career guidance can also provide space to discuss occupational uncertainty and critically examine assumptions about technological change.

Institutions and system designers can support professional agency through the conditions surrounding AI use. Interfaces can present alternatives, disclose uncertainty, explain the basis of recommendations, invite modification, and preserve a record of human decisions. Institutional policy should clarify data governance, acceptable dependence, authorship, responsibility, and the limits of automated decision making. These design and policy measures offer approaches for future evaluation, particularly where concerns about employment and professional status extend beyond individual attitudes.

### 5.4 Limitations and future directions

Several limitations qualify these findings. First, the study captured short-term responses to a single task, not stable changes in professional identity. Baseline adjustment and bootstrap intervals do not resolve this limitation. Longitudinal and practicum-based studies should measure appraisals, mediators, and identity at separate occasions to examine whether repeated GenAI encounters accumulate into durable change. Second, participants were nested within teacher-education programs and institutions, but the analyses were conducted at the individual level. Multilevel research should examine how institutional AI policy, teacher-educator modeling, practicum climate, and labor-market messages shape individual appraisals. The study also relied on a single teaching-design topic, and replication across subject domains and task types is needed. Future experiments should separate capability cues from role-boundary cues and incorporate behavioral indicators of how participants adopt, revise, or reject AI suggestions. Cross-cultural comparisons are also needed because teacher authority, occupational security, and expectations of human care vary across educational systems. Finally, research should examine whether AI literacy, professional calling, practicum experience, perceived employability, institutional support, and prior success with AI account for further variation in these appraisals.

## 6 Conclusion

Perceived GenAI teaching intelligence was associated with pre-service teachers' immediate professional identity through two oppositely signed indirect pathways: a positive association through professional agency and a negative association through replacement threat. At higher levels of teacher–AI complementarity beliefs, the agency-related association was stronger and the threat-related association was less negative. These findings suggest that the educational significance of GenAI involves both perceived capability and its relationship to human judgment, responsibility, and professional value. Teacher education should develop the capacity to work with AI alongside clarity about the professional responsibilities that accompany its use.

## Data Availability

The original contributions presented in the study are included in the article/Supplementary material, further inquiries can be directed to the corresponding author.
