# Supplementary material for: Perceived generative AI teaching intelligence and pre-service teachers' professional identity: double-edged pathways through professional agency and AI replacement threat
Source: Front Psychol. 2026 Sep 28;17:1953782. doi: 10.3389/fpsyg.2026.1953782 (PMC13655131; doi:10.3389/fpsyg.2026.1953782)
Supplement: Supplementary file 1 [file Supplementary_File_1.pdf]

Perceived Generative AI Teaching Intelligence and Pre-Service Teachers' Professional Identity: Double-Edged Pathways Through Professional Agency and AI Replacement Threat

Haoyu Zhao
Hubei Communications Research Institute, Hubei Communications Technical College, Wuhan, China

Supplementary Material

Supplementary Material S1: High- and low-intelligence GenAI teaching-support materials

S1.1 Teaching-design task instruction

Participants were asked to design a 40-minute lesson for Grade 7 students on the topic "effective learning strategies." The teaching design was required to include six parts: learner difficulty analysis, student-centered instructional objectives, teaching procedures and classroom interaction, learning activities, formative assessment and feedback, and a brief explanation of teachers' pedagogical responsibility. Participants were told that the AI teaching-assistant output could be used as a reference, but the final teaching design should reflect their own professional judgment.

S1.2 Prompt used to generate GenAI teaching-support materials

Prompt: You are an AI teaching assistant. Please provide teaching-support suggestions for a 40-minute Grade 7 lesson on "effective learning strategies." Your response should include learner difficulty analysis, student-centered instructional objectives, teaching procedures and classroom interaction, learning activities, formative assessment and feedback, and a brief explanation of how the lesson reflects teachers' pedagogical responsibility. Please provide suggestions that can help a pre-service teacher think professionally rather than simply copy a lesson plan.

S1.3 High-intelligence GenAI teaching-support material

Learner difficulty analysis: Grade 7 students may know general slogans such as "listen carefully" or "review after class," but they often lack operational strategies for planning, monitoring, and evaluating their own learning. Some students may equate effort with long study time and may not know how to select different strategies for different learning tasks.

Instructional objectives: By the end of the lesson, students should be able to (a) explain the meaning of planning, monitoring, and reflection in learning; (b) identify at least three effective learning strategies; (c) select appropriate strategies for a given learning problem; and (d) write a short personal learning-improvement plan.

Teaching procedures: The lesson begins with a short case in which a student spends a long time studying but achieves limited improvement. Students first diagnose the case individually and then discuss in pairs which learning behaviors are ineffective.

The teacher then introduces three strategy groups: goal-setting and planning, active processing of information, and reflection after practice. Students apply these strategies to two classroom scenarios and compare their choices.

Classroom activities: Activity 1 asks students to classify ineffective and effective learning behaviors. Activity 2 uses a think-pair-share format to redesign the study plan of the case student. Activity 3 requires students to complete a "strategy-choice card" for one subject they find difficult.

Feedback and assessment: The teacher collects exit tickets asking students to state one strategy they will try in the coming week and why it fits their learning difficulty. Feedback focuses on whether students can connect the strategy to a specific learning problem rather than simply repeat the strategy name. The teacher may use students' exit tickets to plan follow-up guidance.

S1.4 Low-intelligence GenAI teaching-support material

Learner difficulty analysis: Students may have some problems in learning and may not know enough learning methods. Teachers should help students improve their learning. Instructional

objectives: Students should understand effective learning strategies and know that learning strategies are important. Students should learn some methods and use them in study. Teaching procedures: First, introduce the topic of effective learning strategies. Second, explain several learning methods. Third, ask students to discuss learning strategies in groups. Fourth, ask students to share their ideas. Finally, summarize the lesson and tell students to use good learning methods after class. Classroom activities: Teachers can ask questions, organize group discussion, and let students talk about their learning experience. Students can write down some learning methods. Feedback and assessment: Teachers can ask students whether they understand the lesson and give comments on their answers. Teachers can encourage students to use learning strategies in the future.

S1.5 Manipulation-control considerations

The two materials used the same teaching topic, task requirements, response structure, and classroom duration. The high-intelligence material differed from the low-intelligence material in pedagogical specificity, contextual sensitivity, learner-difficulty diagnosis, instructional sequencing, and feedback relevance. Expert review and a pilot manipulation check were used to ensure that the difference between the two materials reflected teaching-related intelligence rather than a change in topic or task requirement.

Supplementary Material S2: Measurement of control variables

Control variables were included to reduce the possibility that the observed relationships were attributable to demographic background, prior exposure to GenAI, general AI competence, or pre-existing professional identity. Gender, grade level, prior GenAI experience, perceived AI literacy, and baseline professional identity were included in the structural analyses. Teacher-education major was recorded to characterize the sample but was not included in the focal structural model.

Supplementary Table S1. *Control-variable measurement and coding*

Control variable	Coding / item	Use in analysis
Gender	0 = male; 1 = female	Included as a demographic control.
Grade level	1 = first-year; 2 = second-year; 3 = third-year; 4 = fourth-year or above	Included to control for professional-development stage.
Major	Teacher education major reported by participants, such as primary education, preschool education, language education, mathematics education, science education, or educational technology	Recorded for sample description; not included in the focal structural model.
Prior GenAI experience	How frequently have you used GenAI tools for learning or teaching-related tasks? 1 = never; 5 = very frequently	Included to account for prior exposure to GenAI.
Perceived AI literacy	AIL1: I understand the basic functions and limitations of GenAI tools.<br>AIL2: I can evaluate whether AI-generated information is reliable and appropriate.<br>AIL3: I can use GenAI tools appropriately for learning or teaching-related tasks.	Included to distinguish teacher–AI complementarity beliefs from general AI competence.
Baseline teacher professional identity	The same five teacher-professional-identity items were administered before the teaching-design task.	Included to control for pre-existing professional identity.

Note. GenAI = generative artificial intelligence; AIL = perceived AI literacy.

Supplementary Material S3: Supplementary statistical analyses

Supplementary Table S2. Group differences by experimental condition

Variable	High-intelligence condition (n = 302)	Low-intelligence condition (n = 296)	t	p	Cohen's d
PIGT	3.884±0.722	2.937±0.702	16.271	<0.001	1.331
TPA	3.696±0.731	3.427±0.677	4.666	<0.001	0.382

Variable	High-intelligence condition (n = 302)	Low-intelligence condition (n = 296)	t	p	Cohen's d
ART	3.111±0.819	2.884±0.752	3.522	<0.001	0.288
Post-task TPI	**3.870±0.634**	3.795±0.615	1.467	0.143	0.120

*Note. Values are **means ± standard deviations**. PIGT = perceived intelligence of GenAI in teaching; TPA = teacher professional agency; ART = AI replacement threat; TPI = teacher professional identity.*

Supplementary Table S3. Effect sizes of the focal regression paths

Path / endogenous construct	f^2	Interpretation
PIGT → TPA	0.109	Small
PIGT → ART	0.084	Small
TPA → TPI	0.258	Medium
ART → TPI	0.118	Small
TACB → TPA	0.131	Small
TACB → ART	0.121	Small
PIGT × TACB → TPA	0.026	Small
PIGT × TACB → ART	0.014	Negligible / very small
PIGT → TPI (direct association)	0.001	Negligible

Note. f^2 values were computed from the ordinary least squares regression equations as the squared semipartial contribution of each path (t^2/df). Conventional benchmarks are 0.02 (small), 0.15 (medium), and 0.35 (large); values below 0.02 are described as negligible or very small. The PIGT × TACB → ART interaction was statistically significant but locally small.

Supplementary Table S4. Alternative-model sensitivity analyses

Model	Outcome-equation specification	R^2 post-task TPI
Proposed model	Post-task TPI regressed on PIGT, TPA, and ART	0.311
Direct-only benchmark	Post-task TPI regressed on PIGT alone	0.013
